# Supplementary material for: The emergence of moral alignment within human groups is facilitated by interbrain synchrony
Source: Commun Biol. 2025 Mar 20;8:464. doi: 10.1038/s42003-025-07831-4 (PMC11926081; doi:10.1038/s42003-025-07831-4)
Supplement: Supplementary file 1 — Supplementary Material [file 42003_2025_7831_MOESM1_ESM.pdf]

Supplementary Materials

1. Supplementary Methods.

1.1. Group Dominance Structure

The measure is based on subjective assessments independent judges (trained research assistants) provide. In the study, participants engaged in ten deliberation phases. Each deliberation phase was observed by two trained research assistants, who after the end of any deliberation phase indicated (independently) which subject they thought was the most dominant one. As a result, in each deliberation phase, we obtained two independent judgments indicating which subject was the most dominant, which sums up to twenty judgments over the ten deliberation phases. Those twenty judgments were treated as points, such that a subject would gather a point for each time they were selected as the dominant person. This coding scheme results in subjects receiving a 'dominance score' that ranges from zero (never selected as dominant) to twenty (always selected as dominant by both judges). The four subjects in any group would therefore split the twenty potential points between them. To obtain our group dominance structure, we simply calculate the variance in the dominance scores of group members. Lower variance indicates that dominance scores were similar, which resembles an egalitarian group structure, whereas higher variance indicates that dominance scores were very different which resembles a centralized dominance structure.

Supplementary Figure 1.

Enemy territory:

Baxter is the leader of a small group of soldiers. He and his soldiers are on their way back from a completed mission deep in enemy territory when Chris, one of Baxter's men has stepped in a trap that has been set by the enemy and is badly injured. The trap is connected to a radio device that by now has alerted the enemy to the soldier's presence. The enemy will soon be on their way. If the enemy finds the injured man, they will torture him and kill him. He begs Baxter not to leave him behind, but if Baxter tries to take him with him, the entire group will be captured. The only way to prevent this injured soldier from being tortured is for Baxter to shoot him himself.

Is it morally appropriate for Baxter to shoot this soldier in order to prevent him from being tortured by the enemy?

1	2	3	4	5	6	7	8
Completely morally inappropriate	Morally inappropriate	Somewhat immoral	Slightly immoral	Slightly moral	Somewhat moral	morally appropriate	Completely morally appropriate

Example of a Moral Dilemma. A screenshot of a computer screen in which one dilemma (out of ten) is presented to the participants in our study, including the 8-point Likert rating scale. Note that the content in the screen is translated here to English, whereas the actual content in our study was in either Hebrew or Arabic.

Supplementary Table 1.

Models Comparison – The Effect of Condition (Pre-deliberation compared to post-deliberation) on Variance in Private Ratings

Model	N Parameters	BIC	Chi Square	DF	P
Unconditional means	3	4455.9			
Random intercept	4	4184.8	277.97	1	< 2.2e-16 ***
Random slope	5	4191.3	0.3738	1	0.5409

Supplementary Table 2.

Model Comparison – The Effect of Condition (Pre-deliberation compared to post-deliberation) on Deviation of Private Ratings from Consensus

Model	N parameters	BIC	Chi Square	DF	P
Unconditional means	3	4775.8			
Random Intercept	4	4386.6	396.03	1	< 2.2e-16 ***
Random Slope	6	4399.6	0.7365	2	0.692

Supplementary Table 3.

Contrast Analysis for HHb interbrain synchrony Levels between real and pseudo groups across interbrain ROI pairs

ROI	beta	SE	df	t-ratio	p-value
Left dlPFC	-0.0067302	0.001998891	581	-3.367009	0.0008
Right dlPFC	-0.0078238	0.001995608	581	-3.920515	<.0001
Left IFG	-0.0061052	0.001987004	581	-3.072589	0.0022
Right IFG	-0.0098471	0.002007444	581	-4.905315	<.0001
Left pre-motor cortex	-0.0086849	0.002007444	581	-4.326380	<.0001
Right pre-motor cortex	-0.0036198	0.001995608	581	-1.813891	0.0702

Note. This table summarizes the results of the contrasts comparing interbrain synchrony (IBS) values for the real groups compared to the pseudo (real minus pseudo) groups across various interbrain ROI pairs, including the estimate of beta (IBS for real groups minus IBS for pseudo groups), standard error (SE), degrees of freedom (df), t-ratio, and tukey corrected p-value. The results were obtained using the *emmeans()* function in R software provided with the linear regression model as an argument.

Supplementary Table 4.

Marginal means estimates of HHb interbrain synchrony values for all interbrain ROI pairs for

both real and pseudo groups

ROI	Group Type	Mean	SE	df	Lower CL	Upper CL
Left dlPFC	Pseudo	0.3358690	0.001347652	581	0.3332221	0.3385159
	Real	0.3425993	0.001476279	581	0.3396998	0.3454988
Right dlPFC	Pseudo	0.3343939	0.001360306	581	0.3317222	0.3370656
	Real	0.3422177	0.001460144	581	0.3393499	0.3450855
Left IFG	Pseudo	0.3297381	0.001347652	581	0.3270912	0.3323850
	Real	0.3358433	0.001460144	581	0.3329755	0.3387111
Right IFG	Pseudo	0.3272731	0.001360306	581	0.3246014	0.3299449
	Real	0.3371203	0.001476279	581	0.3342208	0.3400198
Left pre-motor cortex	Pseudo	0.3311873	0.001360306	581	0.3285156	0.3338591
	Real	0.3398723	0.001476279	581	0.3369728	0.3427718
Right pre-motor cortex	Pseudo	0.3348488	0.001360306	581	0.3321771	0.3375205
	Real	0.3384686	0.001460144	581	0.3356008	0.3413364

Note. The results were obtained using the *emmeans()* function in R software provided with the linear regression model as an argument. CI represents 95% standard deviation confidence interval.

Supplementary Table 5.

ANOVA Results for the linear regression model

**Source**	**DF**	**SS**	**MS**	**F value**	**p-value**
Group Type	1	0.0047	0.0047	50.0275	<.0001
Interbrain Edge	5	0.0081	0.00162	17.0361	<.0001
Interaction (Group Type * Interbrain Edge)	5	0.000	0.000	0.1027	= 0.9916

Note. The results were obtained using the *anova()* function in R software provided with the linear regression model as an argument. Predicted variable is interbrain synchrony (O2Hb signal).

Supplementary Table 6.

Random Intercept HLM Model – Effect of HHb Interbrain Coupling on Group Moral Alignment Across all Interbrain ROI pairings.

ROI pairing	Term	Mean (SE)	t (df)	P
Left IFG	Intercept	-0.022 (1.77)	-0.013 (357.7)	0.99
	Slope	10.47 (5.29)	1.98 (363.7)	0.048
Right IFG	Intercept	4.516 (1.8)	2.5 (346.8)	0.013
	Slope	-3.07 (5.36)	-0.573 (352.9)	0.5670
Left DLPFC	Intercept	6.723 (1.89)	3.55 (306.5)	<0.001
	Slope	-9.5 (5.52)	-1.719 (312.23)	0.086
Right DLPFC	Intercept	2.46 (1.79)	1.37 (293.93)	0.172
	Slope	3.0 (5.24)	0.574 (300)	0.567
Left PMC	Intercept	4.1 (1.26)	3.23 (410.2)	0.001
	Slope	-1.8 (3.694)	-0.488 (420)	0.625
Left PMC	Intercept	3.11 (1.23)	2.52 (424.08)	0.012
	Slope	1.11 (3.62)	0.3 (432.26)	0.759

Supplementary Table 7.

Random Intercept HLM Model – Effect of O2Hb Interbrain Coupling on Group Moral Alignment Across all Interbrain ROI pairings.

ROI pairing	Term	Mean (SE)	t (df)	P
Left IFG	Intercept	1.19 (1.84)	0.645 (380.6)	0.52
	Slope	6.84 (5.467)	1.25 (385.9)	0.21
Right IFG	Intercept	0.8 (1.83)	0.437 (315.8)	0.662
	Slope	8.04 (5.45)	1.474 (321.1)	0.141
Left DLPFC	Intercept	3.33 (1.8)	1.84 (357.3)	0.065
	Slope	0.43 (5.24)	0.08 (363.8)	0.933
Right DLPFC	Intercept	4.47 (1.9)	2.357 (319)	0.019
	Slope	-2.9 (5.55)	-0.523 (325.2)	0.6
Left PMC	Intercept	3.56 (1.32)	2.69 (424.8)	0.007
	Slope	-0.242 (3.9)	-0.062(431.8)	0.95
Left PMC	Intercept	5.11 (1.2)	4.23 (408.8)	< 0.00
	Slope	-4.74 (3.44)	-1.36 (420.2)	0.174

Supplementary Table 8.

MNI Coordinates of Optodes Used in the Study

Optode	X	Y	Z
Source 1	95.017	112.669	220.931
Source 2	51.2228	143.688	178.093
Source 3	56.6909	113.886	178.853
Source 4	69.2998	85.3373	167.288
Source 5	43.505	114.719	113.312
Source 6	157.062	112.376	224.309
Source 7	189.578	88.2403	171.22
Source 8	203.669	116.032	179.608
Source 9	208.277	144.969	182.624
Source 10	215.495	117.031	120.375
Detector 1	69.4561	127.383	205.522
Detector 2	78.0341	97.6417	195.546
Detector 3	44.0591	131.766	146.243
Detector 4	50.6288	99.6887	143.125
Detector 5	181.795	101.158	197.608
Detector 6	188.997	128.438	206.936
Detector 7	209.84	103.001	148.031
Detector 8	213.495	131.5	152.188

Note. This table presents the Montreal Neurological Institute (MNI) coordinates (X, Y, Z) for each optode (sources 1–10, and detectors 1-8) incuded in the used brite 24 montage. The coordinates define the spatial placement of optodes in millimeters relative to the standardized MNI brain space.

Supplementary Table 9.

10-20 *Reference Points* ***Coordinates***

Electrode	X	Y	Z
Nz	131.1	188	234
Iz	128	182	25.9

RPA	42.9	192	128
LPA	214.1	192	131
Cz	131	46.9	133
Fpz	131	150	235.1
AFz	131	110.2	227.2
Fz	131	79	204.1
FCz	131	56.9	169
CPz	129.5	47.4	94.1
Pz	129	67.4	60.5
POz	128	101.7	40.1
Oz	128	141.1	26.5
T7	215	153	132.1
C5	212.1	115	132
C3	196.6	81.5	133
C1	166.1	57	133
C2	94.4	55.1	131
C4	64	78.9	130
C6	47.5	112	129
T8	43.9	151	128
N1	157	189	225.1
AF9	180.5	192	211.1
F9	200	192	190.1
FT9	214.5	192	165
TP9	219.1	191	100.9
P9	206.1	189	75
PO9	188.5	187.1	48.6
I1	161.3	184	31.1
N2	104.9	189	223.1
AF10	77	192	214.1
F10	57.5	192	188.6
FT10	42.5	192	162

TP10	38.9	190.5	100
P10	54.8	188.5	73.2
PO10	69.9	186	48
I2	96.9	184	31
Fp1	159.8	152.4	230.1
AF7	185	153	217.1
F7	201.9	153	192.3
FT7	212	153	163
TP7	215.8	150.8	105.2
P7	203.1	149	74
PO7	187.7	146.5	48.6
O1	161.1	143	32
Fp2	102	151	231.6
AF8	73.9	152	215
F8	56.9	152	188
FT8	46.9	151	160
TP8	42.4	149.5	99.1
P8	54.8	147	71.2
PO8	71.9	145	47
O2	96.8	142.5	32
AF3	165.9	123.5	224.5
AF4	95.5	122.4	224.1
F5	196	128	196.1
F3	180.1	104	200
F1	160.1	87	203
F6	63.1	125.7	192.6
F4	78	100.9	197
F2	103	83	201.1
FC5	209.1	120	165
FC3	191.7	90.6	167.5
FC1	164.3	66.1	168.6

FC6	49.7	116.9	162.3
FC4	68	87.9	164
FC2	96	62.9	167
CP5	213.1	114	101.5
CP3	196.1	81	98
CP1	168.2	56	95
CP6	48	114.9	97
CP4	64.1	80.7	95
CP2	92	55.9	94
P5	198	118.9	69
P3	185	91	65.9
P1	160.2	70.5	62.5
P6	60.4	119.9	66.5
P4	73	93.9	64
P2	97	76	60.9
PO3	168	113	43.9
PO4	91.5	116.4	40.9

Note. This table presents the Montreal Neurological Institute (MNI) coordinates (X, Y, Z) for each refrence point of the 10-20 system.

Supplementary Table 10.

Source and Detector Pairs (Channel) Comprising Each ROI

**ROI**	**Channel Number**	**Source Number**	**Detector Number**
Right Premotor Cortex	1	4	4
Left Premotor Cortex	2	7	7
Right IFG	3	3	3
Right IFG	4	2	3
Right IFG	5	3	1
Right IFG	6	2	1

Left IFG	7	8	8
Left IFG	8	9	8
Left IFG	9	9	6
Left IFG	10	8	6
Right dlPFC	11	4	2
Right dlPFC	12	1	1
Right dlPFC	13	1	2
Left dlPFC	14	7	5
Left dlPFC	15	6	5
Left dlPFC	16	6	6

Note. This table outlines the channels corresponding to each region of interest (ROI), defined by specific source-detector pairings. Each row identifies a channel number, along with the associated source and detector numbers, for regions including the right and left premotor cortex, inferior frontal gyrus (IFG), and dorsolateral prefrontal cortex (dlPFC).
