# Supplementary material for: The emergence of moral alignment within human groups is facilitated by interbrain synchrony
Source: Commun Biol. 2025 Mar 20;8:464. doi: 10.1038/s42003-025-07831-4 (PMC11926081; doi:10.1038/s42003-025-07831-4)
Supplement: Supplementary file 2 — Description of Additional Supplementary Files [file 42003_2025_7831_MOESM2_ESM.pdf]

Description of Additional Supplementary Files

File name: Supplementary Data 1

Description: This file includes an Excel sheet, referenced in the main manuscript as Supplementary Data 1. It contains the scalp coupling index (SCI) parameter calculations, including the computed SCI values for each participant.
