# Supplementary material for: The emergence of moral alignment within human groups is facilitated by interbrain synchrony
Source: Commun Biol. 2025 Mar 20;8:464. doi: 10.1038/s42003-025-07831-4 (PMC11926081; doi:10.1038/s42003-025-07831-4)
Supplement: Supplementary file 4 — Reporting Summary [file 42003_2025_7831_MOESM4_ESM.pdf]

Corresponding author(s): Aial Sobeh
Last updated by author(s): 2025/2/16

Reporting Summary

Nature Portfolio wishes to improve the reproducibility of the work that we publish. This form provides structure for consistency and transparency in reporting. For further information on Nature Portfolio policies, see our Editorial Policies and the Editorial Policy Checklist.

Statistics

For all statistical analyses, confirm that the following items are present in the figure legend, table legend, main text, or Methods section.

n/a	Confirmed	
☐	☒	The exact sample size (*n*) for each experimental group/condition, given as a discrete number and unit of measurement
☐	☒	A statement on whether measurements were taken from distinct samples or whether the same sample was measured repeatedly
☐	☒	The statistical test(s) used AND whether they are one- or two-sided<br>*Only common tests should be described solely by name; describe more complex techniques in the Methods section.*
☐	☒	A description of all covariates tested
☐	☒	A description of any assumptions or corrections, such as tests of normality and adjustment for multiple comparisons
☐	☒	A full description of the statistical parameters including central tendency (e.g. means) or other basic estimates (e.g. regression coefficient) AND variation (e.g. standard deviation) or associated estimates of uncertainty (e.g. confidence intervals)
☐	☒	For null hypothesis testing, the test statistic (e.g. *F*, *t*, *r*) with confidence intervals, effect sizes, degrees of freedom and *P* value noted<br>*Give P values as exact values whenever suitable.*
☒	☐	For Bayesian analysis, information on the choice of priors and Markov chain Monte Carlo settings
☐	☒	For hierarchical and complex designs, identification of the appropriate level for tests and full reporting of outcomes
☐	☒	Estimates of effect sizes (e.g. Cohen's *d*, Pearson's *r*), indicating how they were calculated

Our web collection on statistics for biologists contains articles on many of the points above.

Software and code

Policy information about availability of computer code

Data collection	No costume code was used for data collection.
Data analysis	All costume code for data analysis are publicly available (doi.org/10.17632/ks8c66myft.1). Data analysis was conducted using R studio.

For manuscripts utilizing custom algorithms or software that are central to the research but not yet described in published literature, software must be made available to editors and reviewers. We strongly encourage code deposition in a community repository (e.g. GitHub). See the Nature Portfolio guidelines for submitting code & software for further information.

Data

Policy information about availability of data

All manuscripts must include a data availability statement. This statement should provide the following information, where applicable:
- Accession codes, unique identifiers, or web links for publicly available datasets
- A description of any restrictions on data availability
- For clinical datasets or third party data, please ensure that the statement adheres to our policy

All primary datasets and referenced datasets are publicly available (doi.org/10.17632/ks8c66myft.1)

Research involving human participants, their data, or biological material

Policy information about studies with human participants or human data. See also policy information about sex, gender (identity/presentation), and sexual orientation and race, ethnicity and racism.

Reporting on sex and gender	Information about participants' gender was collected through a self-report question included in the demographic questionnaire. Out of 188 participants, 133 identified as female, the rest as male.<br>Participants were randomly assigned into groups of four. Members of each group did not necessarily share the same gender. As a result, we had groups of mixed genders and groups of the same gender (all female or all male).<br>We treated the variable group gender composition as a binary variable (mixed-groups vs. same gender groups). An analysis that included this variable is reported in section 3.3. of the study.
Reporting on race, ethnicity, or other socially relevant groupings	Participants were either natively Arabic-speaking or Hebrew-speaking. Information about race, ethnicity, religion, or socioeconomic status was not collected.<br>Participants were randomly assigned into groups of four participants per group. Participants within each group shared the same native language, either Hebrew or Arabic (N Arabic = 34 groups), ensuring the study was conducted in their mother tongue for optimal understanding of the material and effective expression of their opinions.
Population characteristics	See Above
Recruitment	Participants were students at the University of Haifa recruited by responding to ads on campus or through social media. General exclusion criteria included left handedness, reading difficulties and any history of neurological or psychiatric disorders.
Ethics oversight	The study was approved by the local Ethics Committee of the University of Haifa, and all participants signed an informed consent form prior to being admitted to the study. All ethical regulations relevant to human research participants were followed.

Note that full information on the approval of the study protocol must also be provided in the manuscript.

Field-specific reporting

Please select the one below that is the best fit for your research. If you are not sure, read the appropriate sections before making your selection.

☐ Life sciences ☒ Behavioural & social sciences ☐ Ecological, evolutionary & environmental sciences

For a reference copy of the document with all sections, see nature.com/documents/nr-reporting-summary-flat.pdf

Behavioural & social sciences study design

All studies must disclose on these points even when the disclosure is negative.

Study description	The study has a correlational research design testing the relationship between quantitative measures.
Research sample	Participants were students at the University of Haifa. Of 188 participants (in the final sample), 133 identified as female, the rest as male. (Age: Mean: 22.5, Range: 19 - 36).
Sampling strategy	Participants were students at the University of Haifa recruited by responding to ads on campus or through social media (Convenience sampling). Initial sample size included a total of 200 participants who were randomly assigned to 50 groups of four participants per group. The desired sample size (groups n) was calculated using power analysis carried out in G*Power 3.1 (46), with an assumed moderate effect size, $\alpha = 0.05$, and power of 0.80 (47) to ensure detection of significant effects in our main regression model.
Data collection	The data collected both behavioral and neural activity measures. Raw behavioral data was collected through a computerized task. Raw neural data was collected using functional near-infrared spectroscopy (fNIRS) devices. No one besides two researchers and the participants was present during the study. The researchers were not blind to the research hypothesis during data collection.
Timing	Start date: November 18, 2021<br>End date: January 19, 2023
Data exclusions	A total of 200 participants were initially recruited and randomly assigned to 50 groups of four participants per group. Three groups were later excluded from the analysis due to data acquisition problems (final sample: 47 groups, participants = 188, female=133).
Non-participation	Of participants who were invited to participate, no one dropped out or were declined participation.<br>Some participants were not invited to participate based on whether they met one or more of the exclusion criteria (reading difficulties, left-handedness, and a history of neurological and/or psychiatric disorder).
Randomization	The study did not include experimental groups.

Reporting for specific materials, systems and methods

We require information from authors about some types of materials, experimental systems and methods used in many studies. Here, indicate whether each material, system or method listed is relevant to your study. If you are not sure if a list item applies to your research, read the appropriate section before selecting a response.

Materials & experimental systems

n/a	Involved in the study
☒	☐ Antibodies
☒	☐ Eukaryotic cell lines
☒	☐ Palaeontology and archaeology
☒	☐ Animals and other organisms
☒	☐ Clinical data
☒	☐ Dual use research of concern
☒	☐ Plants

Methods

n/a	Involved in the study
☒	☐ ChIP-seq
☒	☐ Flow cytometry
☒	☐ MRI-based neuroimaging

Plants

Seed stocks	*Report on the source of all seed stocks or other plant material used. If applicable, state the seed stock centre and catalogue number. If plant specimens were collected from the field, describe the collection location, date and sampling procedures.*
Novel plant genotypes	*Describe the methods by which all novel plant genotypes were produced. This includes those generated by transgenic approaches, gene editing, chemical/radiation-based mutagenesis and hybridization. For transgenic lines, describe the transformation method, the number of independent lines analyzed and the generation upon which experiments were performed. For gene-edited lines, describe the editor used, the endogenous sequence targeted for editing, the targeting guide RNA sequence (if applicable) and how the editor was applied.*
Authentication	*Describe any authentication procedures for each seed stock used or novel genotype generated. Describe any experiments used to assess the effect of a mutation and, where applicable, how potential secondary effects (e.g. second site T-DNA insertions, mosiacism, off-target gene editing) were examined.*
